# Illuminating the Characteristics and Assembly of Prokaryotic Communities across a pH Gradient in Pit Muds for the Production of Chinese Strong-Flavor Baijiu

**DOI:** 10.3390/foods13081196

**Published:** 2024-04-15

**Authors:** Mingdong Deng, Xiaolong Hu, Yong Zhang, Xinyu Zhang, Haifeng Ni, Danyang Fu, Lei Chi

**Affiliations:** 1 Food Laboratory of Zhongyuan, Henan Key Laboratory of Cold Chain Food Quality and Safety Control, College of Food and Bioengineering, Zhengzhou University of Light Industry, Zhengzhou 450001, China; 2 School of Life Science and Technology, Xi’an Jiaotong University, Xi’an 710048, China

**Keywords:** baijiu, pit mud, physicochemical properties, co-occurrence network, assembly process

## Abstract

Pit mud (PM), as an important source of microorganisms, is necessary for Chinese strong-flavor baijiu (CSFB) production. Although it has been revealed that the PM prokaryotic community diversities are influenced by its quality, product area, ages, etc., the characteristics and assembly process of the prokaryotic community in PMs across a pH gradient are still unclear. In this study, the regular changes of α- and β-diversities of the prokaryotic community across a pH gradient in PMs were revealed, which could be divided into “stable”, “relatively stable”, and “drastically changed“ periods. A total of 27 phyla, 53 classes, and 381 genera were observed in all given samples, dominated by Firmicutes, Bacteroidetes, Proteobacteria, *Lactobacillus*, *Caproiciproducens*, *Proteiniphilum*, etc. Meanwhile, the complexity of the network structure of the prokaryotic microbial communities is significantly influenced by pH. The community assembly was jointly shaped by deterministic and stochastic processes, with stochastic process contributing more. This study was a specialized report on elucidating the characteristics and assembly of PM prokaryotic communities across a pH gradient, and revealed that the diversity and structure of PM prokaryotic communities could be predictable, to some degree, which could contribute to expanding our understanding of prokaryotic communities in PM.

## 1. Introduction

Fermented foods have been a part of the human diet for thousands of years [1] and developed along with human civilization, such as red pepper paste, soy sauce, kimchi, yogurt, bread, etc. [2]. They have served not only as a means of preserving food but also for their distinctive flavors. Chinese baijiu, one of the representative traditional fermented beverages, has been divided into 12 flavor types based on its unique flavors, and Chinese strong-flavor baijiu (CSFB) is one of the most popular types. The solid-state fermentation (SSF) system of CSFB mainly involves components including pit mud (PM), fermented grains (FGs), and Daqu starter. It is a typical semi-natural prokaryotic ecosystem distinguished by its openness and spontaneity [3]. PM is an important microbial carrier and a primary source of aroma substances for the CSFB production. Compared to CSFB produced without PM, the concentrations of the primary aroma compounds in that produced with PM were significantly higher, e.g., acetic acid, butanoic acid, hexanoic acid, ethyl hexanoate, etc. [4]. Therefore, the study of PM has always been a hot topic of the research on CSFB. The complex microbial communities with hundreds of genera in PM, mainly assigned into the phyla of Firmicutes, Bacteroidetes, Proteobacteria, Euryarchaepta, Synergistetes, etc., have been clearly revealed in various PMs with different ages, qualities, and product areas [5,6,7,8]. For instance, the genera including *Caproicibacterium*, *Clostridium*, *Lactobacillus*, and so on, were the most commonly detected bacteria, which significantly contribute to the formation of the flavor compounds in CSFB. Some of these microorganisms have been isolated from PMs, e.g., *Ruminococcaceae* bacterium CPB6, *Caproicibacterium* sp. JNU-WLY1368, and *Clostridium kluyveri* N6 [9,10]. All of these indicate that PM could serve as a valuable reproducible, accessible, and culturable SSF ecosystem model for dissecting the physicochemical driving factors, understanding microbial assembly mechanisms, and investigating the interactions within complex microbial communities.

Previous studies reveal that the diversity, structure, and succession of the prokaryotic communities in PMs were strongly correlated with their ages, qualities, product area, and locations within the fermentation pits [11,12,13]. For instance, the prokaryotic community diversity typically increases with the age, quality, and sampling depth of PM, and the abundance of genus *Lactobacillus* decreases, while the genera assigned into Clsotridia (e.g., *Clostridium*, *Syntrophomonas*, and *Sedimentibacter*) and Bacteroidia (e.g., *Petrimonas* and *Prevotella*) increase [4,14,15]. In fact, the physicochemical properties of the above types of PMs are different, including factors such as pH, moisture content, total acid concentration, ammonium nitrogen, available phosphorus, and more. These properties are considered to be the primary driving forces shaping the microbial communities within PMs. Among them, a low pH value, low contents of ${\mathrm{NH}}_{4}^{+}\;$ and available phosphorus, but high lactate content, would always result in a lower diversity of prokaryotic community and higher content of lactic acid bacteria (especially *Lactobacillus*) [15,16,17]. Therefore, some physicochemical factors, with the advantages of being easy to detect, fast, and of low cost, could be used to preliminarily evaluate the quality and microbial information of PMs. Among them, the pH, lactic acid, dissolved organic carbon (DOC), and ${\mathrm{NH}}_{4}^{+}$ are the main triggers of microbial diversity in PMs [4,15], and pH is significantly correlated to other factors, e.g., DOC, lactate, ${\mathrm{NH}}_{4}^{+},\;$ and available phosphorus [18]. It is similar to the soil [19], and the pH could also be used as a comprehensive indicator to assess the microbial and physicochemical properties of various PMs. However, the changes in physicochemical properties, microbial network complexity, and the prokaryotic community structures and diversities across a pH gradient in PMs are still unclear today.

Moreover, the assembly rules of microbial communities are of increasing interest to ecologists [20]. There are two major processes known to influence how communities are assembled, including niche-based deterministic and neutrality-based stochastic processes [21,22]. The niche-based theory asserts that deterministic processes, including environmental filtering (e.g., pH, temperature, moisture, and salinity) and various biological interactions, largely control the patterns of species composition, abundance, and distributions. However, the neutral theory emphasizes that all species are ecologically equivalent and the assembly process is largely controlled by the stochastic processes (e.g., birth/death, immigration) [23,24]. It is widely accepted that both deterministic and stochastic processes operate simultaneously during community assembly. For instance, the fungal community assembly in wild stoneflies is driven by both deterministic and stochastic forces, and deterministic processes play a larger role than stochasticity [25]. The stochastic and deterministic processes together shape microbial community assemblies in FGs, and their contribution changes with the CSFB fermentation [26]. Therefore, investigating the assembly process of the prokaryotic community in PM and its changes under different pH conditions are beneficial for understanding the highly complex PM ecosystems and their functions. Moreover, it is the first prerequisite for ensuring reproducible fermentation outcomes, as different microbiome assembly patterns can lead to different functional outputs.

In this work, the PMs with a pH gradient, spanning approximately pH 3 to 9, were collected. Then, the effect of pH on the prokaryotic community diversities and other physicochemical factors of PMs were analyzed by using a combination of Illumina HiSeq sequencing, physicochemical analysis, and multivariable statistics methods. Additionally, the relationships between dominant prokaryotic taxa and physicochemical properties, the interaction networks of PM prokaryotic communities, and the process of prokaryotic community assembly were also revealed, respectively. To the best of our knowledge, this is the first specialized study to elucidate the characteristics and assembly of prokaryotic communities across a pH gradient in PMs. Our findings could provide valuable insights for further understanding the PM prokaryotic community and physicochemical properties influenced by pH levels, which is conducive to developing the bioaugmentation technology for improving the PM quality and dynamically monitoring and guiding CSFB production timely.

## 2. Materials and Methods

### 2.1. Sample Collection and Analysis of Physicochemical Properties

As mentioned above, the physicochemical properties of PMs used for the CSFB production are closely correlated with their ages, qualities, sampling depth in the fermentation pit, and geographical situation, so various of types of PMs were collected to obtain more samples with different pHs. In this work, six CSFB enterprises located in the eastern, western, southern, northern, and central parts of Henan Province, China, were respectively selected, and 4 cuboid fermentation pits with different ages or quality grades in each enterprise were selected, and 4 PM samples with different spatial positions (top, middle, under, and bottom layers) in each pit (each pit size about 2.5 m × 1.8 m × 2 m) were respectively collected (Figure S1). Finally, 9 PM samples with white crystals were removed, and a total of 87 PM samples were retained for further analysis. Each PM sample was split into two equal portions, which were stored in an anaerobic bag at −30 °C and then used for physicochemical tests and prokaryotic community analysis by using high-throughput sequencing, respectively.

PM was soaked in deionized water (*w*/*v* ratio, 1:3) and sonicated for 10 min; a supernatant was used for pH. The contents of total acid (TA), moisture (Mo) and ${\mathrm{NH}}_{4}^{+}$ (ammonium nitrogen, AN) of PM samples were detected using the methods described in previous studies [15,27,28]. The available phosphorus (AP) content in PM was detected using the method described in the Agricultural Industry Standard of the People’s Republic of China [29].

### 2.2. DNA Extraction, Amplification, Illumina Sequencing, and Sequence Processing

Total genome DNA from each PM sample was extracted using a HiPure Soil DNA Kit (Magen, Guangzhou, China) according to the manufacturer’s protocols. The upstream primer 5′-CCTACGGRRBGCASCAGKVRVGAAT-3′ and downstream primer 5′-GGACTACNVGGGTWTCTAATCC-3′ were used to amplify the V3 and V4 hypervariable regions of prokaryotic 16S rDNA. PCR reactions were performed in triplicate, each consisting of a 25 μL mixture that contained 2.5 μL of TransStart Buffer, 2 μL of dNTPs, 1 μL of each primer, and 20 ng of template DNA. The indexed adapters were incorporated into the ends of the 16S rDNA amplicons to create indexed libraries. These libraries were then prepared for subsequent next-generation sequencing (NGS) on an Illumina sequencing instrument, following the protocol provided by the manufacturer (Illumina, San Diego, CA, USA). Paired-end sequencing was performed, and the image analysis and base calling were conducted by the Control Software embedded in the instrument.

Double-end sequencing of positive and negative reads was firstly joined together, and the above, merged sequence with base “N” was filtered, while the sequence with a length ≥200 bp was retained. After quality filtering, purifying chimeric sequences, the resulting sequence for operational taxonomic units (OTUs) clustering was analyzed using VSEARCH (1.9.6) (sequence similarity was set to 97%). The representative sequence of each OTU was compared with the 16S rRNA reference database (Silva 132), and then the taxonomic assignments of the above sequences were performed using the RDP (Ribosomal Database Program) classifier Bayesian algorithm. Therefore, each OTU was classified at the phylum, class, order, family, and genus level for further analyzing the community structure under different levels.

### 2.3. Statistical Analysis

The community alpha diversity indices (e.g., Shannon, Chao1, etc.) were calculated based on the results of OTU analysis and data flattening (random sampling the same number of sequences), which aims to accurately reflect the species richness and diversity within the samples. Principal co-ordinates analysis (PCoA) was performed based on Bray–Curtis distances by using the “vegan” package in R software (Version 4.2.1) and visualized by using the “ggplot2” package. Redundancy analysis was visualized by Canno 5. Abundance data were collated and plotted using Origin 2021. Binary regression analysis between major genera and the pH was calculated and visualized using the software SPSS 22 (IBM Corp., Armonk, NY, USA). Phylogenetic information was visualized using the iToL website (https://itol.embl.de, accessed on 3 May 2023). Linear discriminant analysis Effect Size (LEfSe) and the random forest algorithm analyses were calculated by the R packages “randomForest” and “microeco”. Niche width was calculated using the “spaa” package, and specialists were defined as having a niche width of <1.5 and generalists of >3, and the remaining species fall into the normal category.

### 2.4. Microbial Network Construction and Characterization

A network analysis was employed to evaluate the correlations of the prokaryotic taxa with OTUs serving as the nodes in the networks. Only the OTUs that occurred in more than one-sixth of all samples and had an average relative abundance of 0.01% or higher were selected. To ensure the significant and strong relationships of edges, only pairwise correlations with Spearman’s r ≥ 0.6 or ≤ −0.6, and a *p*-value of ≤0.01 were selected for the visualization of network properties. The result was visualized using the Gephi interactive platform (Version 0.9.2).

Based on the criteria used in previous studies, the network hubs (Zi of ≥2.5 and Pi of ≥0.62), module hubs (Zi of ≥2.5 and Pi of <0.62), connectors (Zi of <2.5 and Pi of 0.62), and peripherals (Zi of <2.5 and Pi of <0.62) were identified, respectively. Network hubs are nodes that are highly connected both within and between modules. Module hubs are nodes that are highly connected within a module, and connectors are nodes that are highly connected between modules. All of these nodes can be referred to as keystone taxa [30].

### 2.5. The Phylogenetic-Bin-Based Null Model Analysis

The infer Community Assembly Mechanisms by Phylogenetic-Bin-Based Null Model Analysis (iCAMP, Version 1.5.12) divided the observed taxa into individual bins based on their phylogenetic signal. Then, the process governing each bin is identified based on the null model analysis of the phylogenetic diversity using the beta Net Relatedness Index (βNRI) and taxonomic β-diversities using a modified Raup–Crick metric (RC). For each bin, it would be considered as the homogeneous selection when pairwise comparisons with βNRI < −1.96, and as the heterogeneous selection when pairwise comparisons with βNRI > +1.96. Then, the pairwise comparisons with |βNRI| ≤ 1.96 and RC < −0.95 would be considered as the homogenizing dispersal, while those with |βNRI| ≤ 1.96 and RC > + 0.95 as dispersal limitations. The remains with |βNRI| ≤ 1.96 and |RC| ≤ 0.95 represent the percentages of drift, diversification, weak selection, and weak dispersal, hereafter, simply designated as “drift and others” for convenience [31].

## 3. Results

### 3.1. Physicochemical Properties in PMs across a pH Gradient 

**Figure 1:**
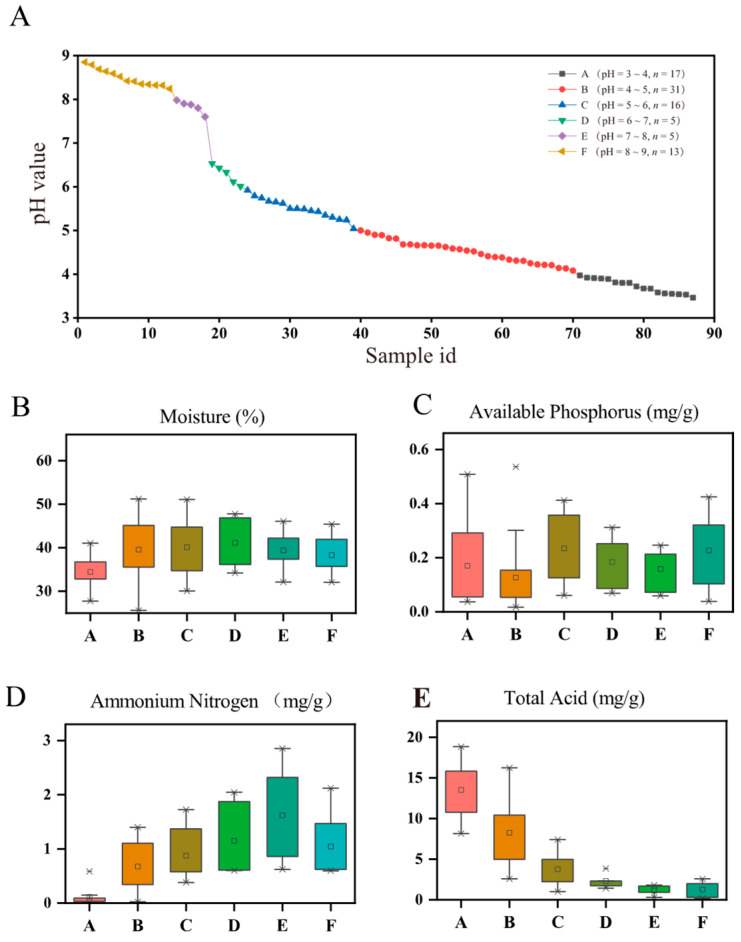
Physicochemical properties of tested PMs. (**A**) Point plot depicting the pH values of PM samples and their respective groupings; changes in (**B**) moisture, (**C**) available phosphorus, (**D**) ammonium nitrogen, and (**E**) total acid across a pH gradient in PMs from groups A to F (pH 3 to 9).

The physicochemical properties of 87 samples were analyzed, with the content of Mo ranging from 25.60% to 51.21%, TA from 0.16 to 18.83 mg/g, AN from 0.01 to 2.85 mg/g, AP from 0.02 to 0.54 mg/g, and pH from 3.46 to 8.85. All samples were divided into 6 groups across a pH gradient, including groups A (4 ≥ pH > 3), B (5 ≥ pH > 4), C (6 ≥ pH > 5), D (7 ≥ pH > 6), E (8 ≥ pH > 7), and F (9 ≥ pH > 8) (Figure 1A). Furthermore, the samples with a pH value more than 8 were considered to be alkaline (i.e., group F) PMs, ones with pH values between 6 and 8 were considered to be near-neutral (i.e., groups D and E) PMs, and the acidic ones (i.e., groups A, B and C) with a pH less than 6.

As shown in Figure 1, except for AP, the above physicochemical factors of PMs showed regular changes with the pH value increasing (from 3.46 to 8.85). Among them, the contents of Mo and AN increased firstly and decreased later, with peaks on groups D and E (Figure 1B,D), respectively. This pattern indicated that there would be a high content of Mo and AN in near-neutral PMs (pH 6–8). The TA contents in PMs showed a downward trend across the observed pH range (Figure 1E). Furthermore, the strong and significant correlations between pH and the other four physicochemical factors were observed by using the Spearman’s correlation analysis, respectively, and the order of correlation with pH value from highest to lowest is TA > AN > AP > Mo (Table S1).

### 3.2. Variation in α- and β-Diversities of Prokaryotic Communities in PMs across a pH Gradient

**Figure 2:**
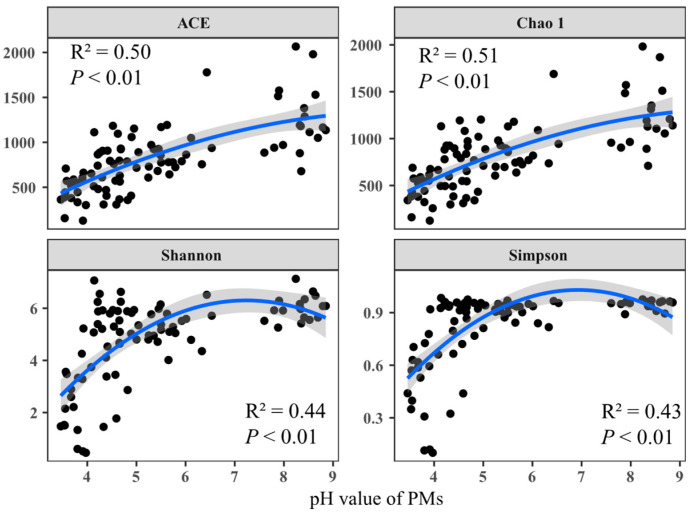
Changes in the alpha diversity indices of prokaryotic communities across a pH gradient in PMs. Each black dot represents a tested PM sample. The blue line is linear regression and 95% confidence interval is in gray. R square and *p* value were marked in figures, respectively.

The prokaryotic communities of 87 tested PMs were analyzed using the Illumina sequencing (Figure S2). For α- diversity of the prokaryotic community, the ACE (from 128.87 to 2065.11), Chao1 (from 125 to 1981.12), Shannon (from 0.45 to 7.13), and Simpson (from 0.10 to 0.98) were detected in all PMs. Additionally, significant correlations were found between pH and the α-diversity indices (*p* < 0.01). Generally, all the mentioned diversity indices increased from acidic to alkaline conditions. The Shannon and Simpson indices, which reflect species diversity, initially increased and then decreased, reaching a peak at approximately pH 7. There was also a notable increase from pH 3 to 5 (Figure 2).

**Figure 3:**
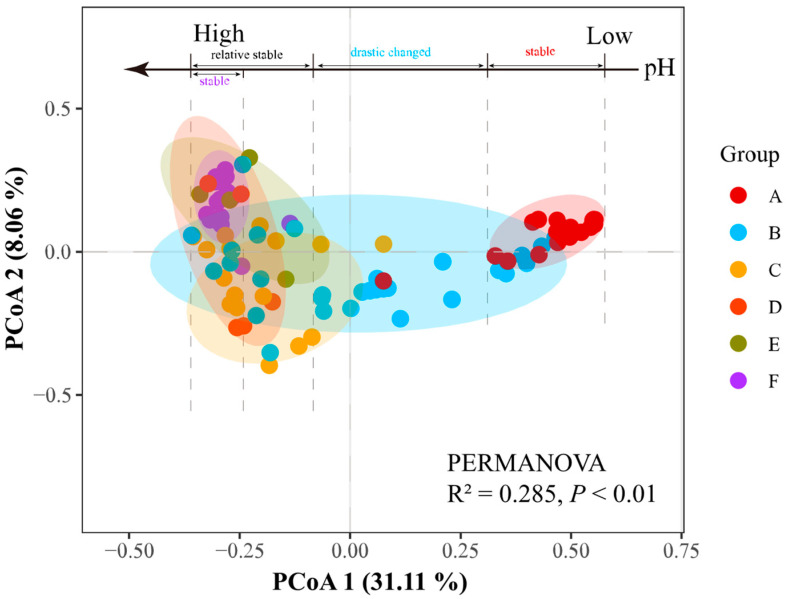
Principal component analysis (PCoA) of prokaryotic communities in PMs assigned into groups A to F based on Bray–Curits distance. Each dot represents a tested PM sample and colored according to pH.

For the β-diversity of prokaryotic communities, the principal co-ordinates analysis (PCoA) and permutational multivariate analysis of variance analysis (PERMANOVA) showed that the prokaryotic compositions in PMs were significantly influenced by their pHs (Figure 3). All of the PMs in group A were located on the right side of the PCoA plot, while most PMs in groups C, D, E, and F were found on the left side. However, the PMs in group B were distributed on both sides. Furthermore, the samples in groups A (pH 3–4) and F (pH 8–9) respectively, exhibited high levels of aggregation. These findings suggested that the prokaryotic communities in PMs exhibited more similar compositions at extreme pH conditions (pH ≤ 4 or pH ≥ 8). Additionally, similar compositions were at a pH from 5 to 8, with a rapid transition occurring between pH 4 and 5.

### 3.3. The Overall Features of Prokaryotic Community Structure in PMs

**Figure 4:**
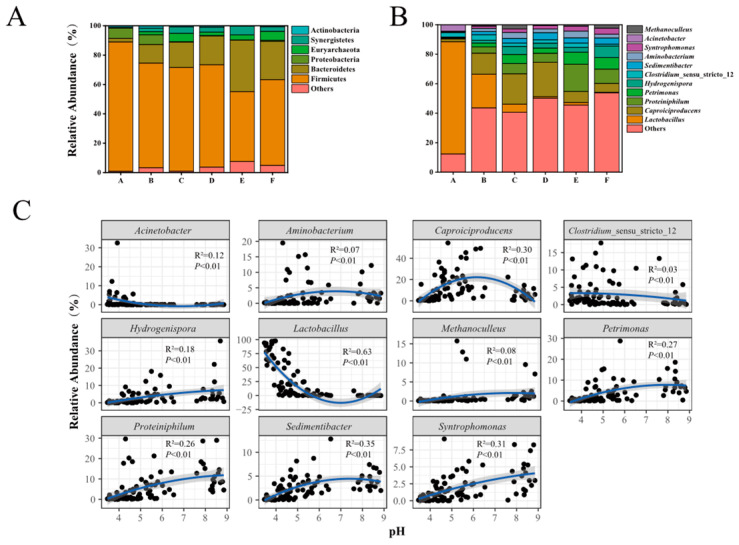
Changes in the prokaryotic community structures across a pH gradient in PMs. (**A**) Relative abundances of prokaryotes in groups A to F of PMs at the phylum level; (**B**) relative abundances of the prokaryotes in PMs at the genus level; (**C**) statistics and linear regression analysis of the correlations between the dominant genera and pH values. Each black dot represents a tested PM sample. The blue line is linear regression and 95% confidence interval is in gray. R square and *p* value were marked in figures, respectively.

For all PM samples, a total of 27 phyla, 53 classes, 381 genera, and unclassified taxon were detected, respectively. At the phylum level, a total of 6 phyla including Firmicutes (mean relative abundance: 71.07%), Bacteroidetes (15.24%), Proteobacteria (3.94%), Euryarchaepta (3.05%), Synergistetes (2.72%), and Actinobacteria (1.09%) were considered as dominant phyla (mean relative abundance > 1%), accounting for 71.08~99.99% in each sample (Figure 4). In addition, five phyla with less abundance (mean relative abundance < 1%), including Cloacimonetes, Tenericutes, Patescibacteria, Chloroflexi, and Cyanobacteria, were observed. At the class level (Figure S4), a total of 7 dominant classes were identified, including Clostridia (44.47%), Bacilli (25.87%), Bacteroidia (15.23%), Gammaproteobacteria (3.21%), Synergistia (2.72%), Methanomicrobia (1.59%), and Methanobacteria (1.27%). At the genus level, a total of 11 dominant genera were identified, including *Lactobacillus* (24.23%), *Caproiciproducens* (11.93%), *Proteiniphilum* (5.79%), *Petrimonas* (3.97%), *Hydrogenispora* (3.31%), *Clostridium*_sensu_stricto_12 (2.85%), *Sedimentibacter* (2.54%), *Aminobacterium* (2.34%), *Syntrophomonas* (1.90%), *Acinetobacter* (1.10%), and *Methanoculleus* (1.05%), accounting for 19.87~99.62% in each sample (Figure 4B and Figure S5). Moreover, a total of 180 rare prokaryotes with a relative abundance of less than 0.01% were observed, including *Methylococcus*, *Syntrophobacter*, *Brevibacillus*, *Kocuria*, etc. In addition, about 16.01% of the total sequences were assigned into unclassified taxa, and their relative abundances presented an upward trend that ranged from 2.98% to 25.06% with pH increasing (Table S2). These findings indicated that PM is a complex microecology system harboring a rich array of microbial resources, including potential novel genetic and species diversity.

As shown in Figure 4, the community structure of PM varied significantly across a pH gradient both at the phylum and genus levels. The relative abundances of Firmicutes and Proteobacteria in PMs decreased with pH increasing, while Bacteroidetes presented an opposite trend. The Euryarchaeota and Synergistetes presented with higher contents when the pH was more than 5. Furthermore, the relative abundances of many (63.64%, 7/11) dominant genera increased from acidic to near-neutral or alkaline PMs (Figure 4B,C, Table S2). Among them, the highest relative abundances of *Petrimonas*, *Proteiniphilum*, *Sedimentibacter*, *Aminobacterium*, and *Caproiciproducens* were observed in near-neutral PMs, and those of *Hydrogenispora* and *Syntrophomonas* were detected in alkaline PMs. However, the relative abundances of three genera, including *Lactobacillus*, *Acinetobacter*, and *Closiridium*_sensu_stricto_12, showed a downward trend from acidic to alkaline PMs, especially the abundances of *Lactobacillus*, which reduced from 76.12% to 0.31%. For the dominant genera, the abundances of *Caproiciproducens* and *Closiridium*_sensu_stricto_12 were more than 1% in all groups of PMs, and those of seven genera were more than 1% in five of all groups of PMs, while those of *Acinetobacter* and *Methanoculleus* were more than 1% only in one or two groups of PMs (Table S2).

### 3.4. Effects of Physicochemical Factors on Prokaryotic Community and Biomarkers in PMs

**Figure 5:**
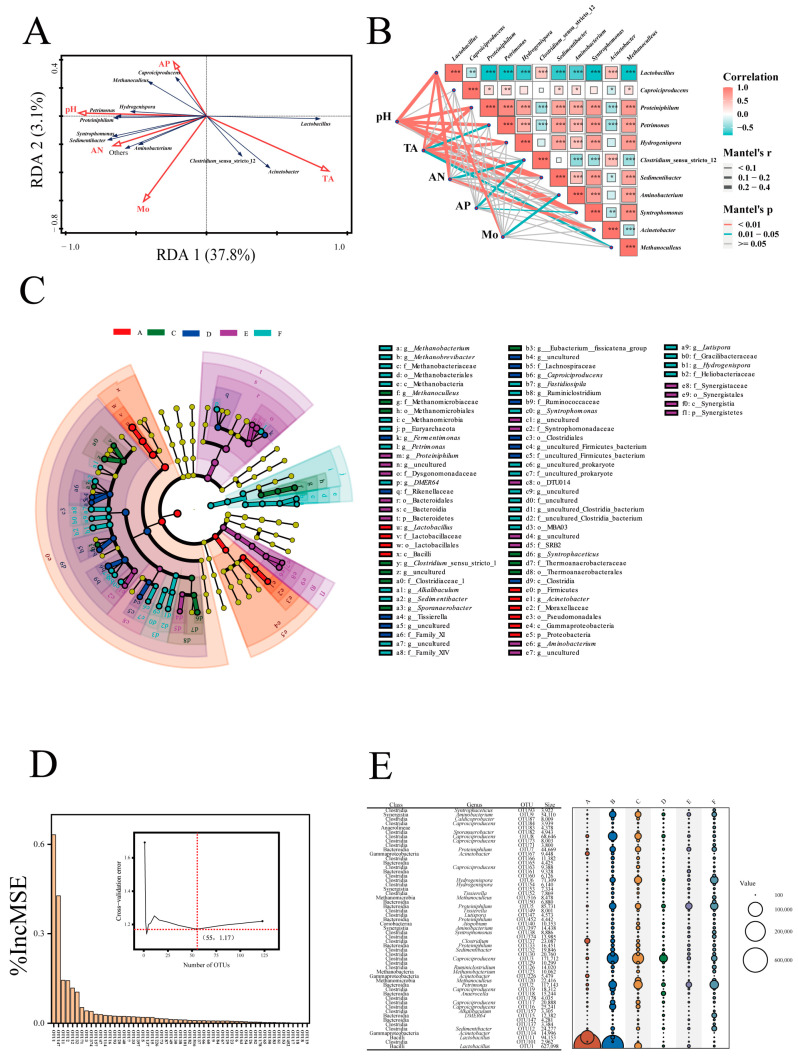
(**A**) Redundancy analysis (RDA) of physicochemical properties and prokaryotic community structure. Arrows indicate the direction and magnitude of measurable variables associated with prokaryotic community structures. (**B**) Mantel test revealing the relationships between physicochemical factors and dominant genera in all samples, respectively. *, *p* < 0.05; **, *p* < 0.01; ***, *p* < 0.001. (**C**) the results of Linear discriminant analysis Effect Size (LEfSe) analysis were presented using tree charts Dots represents genera and colored according to pH. The dots colored in yellow indicate that no signal has been detected; (**D**) the biomarkers that were identified by applying Random Forests machine-learning algorithm and the result of the 10-fold cross-validation method was inserted in the figure; (**E**) bubble diagram showing the relative abundances of biomarkers with different pH groups.

Redundancy analysis (RDA) revealed that only 3 dominant genera (*Lactobacillus*, *Acinetobacter*, and *Closiridium*_sensu_stricto_12) were positively correlated with the TA and negatively correlated with other physicochemical factors (i.e., pH, AN, AP, and Mo), while the other 8 genera (e.g., *Petrimonas*, *Sedimentibacter*, *Caproiciproducens*, and so on) presented an opposite trend (Figure 5A). This finding aligns with the observed changes in dominant genera within PMs as the pH increases (Figure 4C). A Mantel test revealed that pH, compared to other factors, had a considerable impact on the microbial structure of PM, as statistically significant correlations between pH and six genera (Mantel’s r > 0.2, Mantel’s *p* < 0.01) were observed (Figure 5B).

To visualize the significantly different taxa (or biomarkers) of the prokaryotic community in different groups of PMs, the linear discriminant analysis (LEfSe) with a cutoff LDA score of 4.0 (Kruskal–Wallis = 0.05, Wilcoxon = 0.05) were performed (Figure S6). The biomarkers at the kingdom-to-genus levels were displayed in the branch diagram, and 22 biomarkers at the genus level were observed (Figure 5C). Simultaneously, the random forest machine-learning algorithm and 10-fold cross-validation method were performed to evaluate the importance of each OUT (Figure 5D). A total of 55 important OTUs were selected, which originated from 2 kingdoms, 7 phyla, 9 classes, and 22 identified genera (e.g., *Lactobacillus*, *Caproiciproducens,* and so on) (Figure 5E). Based on criteria used in previous studies [3], 18 genera obtained by using the above two methods were considered to be biomarkers, as follows. *Lactobacillus* and *Acinetobacter* for group A, *Methanoculleus*, *Syntrophaceticus*, and *Sporanaerobacter* for group C, *Caproiciproducens* and *Tissierella* for group D, *Aminobacterium* and *Proteiniphilum* for group E, and *Hydrogenispora*, *Petrimonas*, *Sedimentibacter*, *DMER64*, *Syntrophomonas*, *Methanobacterium*, *Lutispora*, *Ruminiclostridium*, and *Alkalibaculum* for group F.

### 3.5. The Network Patterns of Prokaryotic Communities in PMs with Different pHs

**Figure 6:**
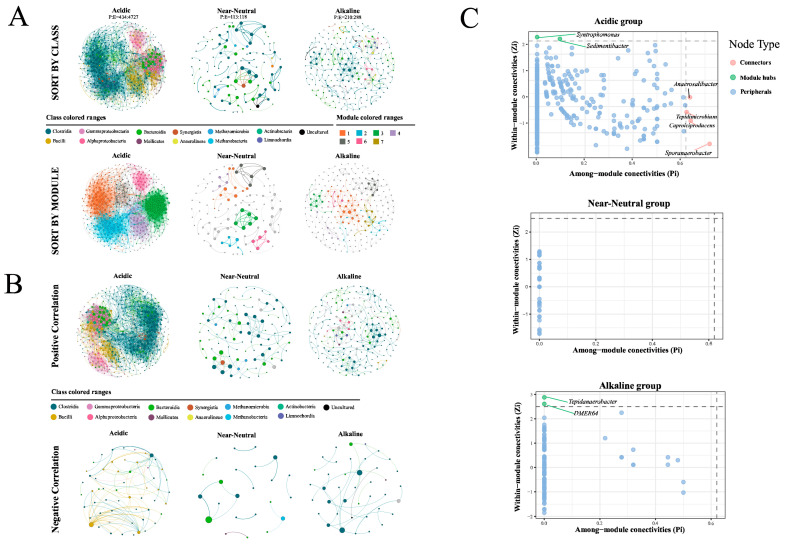
(**A**) The relative networks of prokaryotic communities in acidic, near-neutral, and alkaline PMs sorted by classes and modules; (**B**) the positive and negative relationship networks; (**C**) putative key taxa identified based on their topological roles within networks.

The networks were established based on positive and negative correlation, respectively. As shown in Figure 6 and Table S4, the order of complexities of networks were acidic (nodes/edges = 434/4727) > alkaline (210/288) > near-neutral group (113/118). The ratios of positive to negative correlations were notably high in all successional series. The acidic group had positive and negative edges, accounting for 4643 (98.2%) and 84 (1.8%), respectively. The near-neutral group had 103 positively (87.3%) and 15 (12.7%) negative edges (Figure 6B). The negative correlations within the acidic group were primarily concentrated in nodes assigned to *Lactobacillus* (OTU240 and OTU1) and *Caproiciproducens* (OTU2759), respectively.

Furthermore, all networks of the three above groups of PMs exhibited a certain degree of modularity (each modularity index > 0.44, Table S4) (Figure 6A). A total of 6 dominated modules, accounting for 98.16% of the nodes in the acidic group, were observed (Figure 6A). All modules were comprised of different taxonomic profiles. In the acidic group, Modules 1, 2, and 5 predominantly consisted of Clostridia, Bacteroidia, and Alphaproteobacteria, while Module 3 was mainly composed of Gammaproteobacteria, Module 4 primarily comprised Bacilli, and Module 6 was mainly characterized by Alphaproteobacteria and Gammaproteobacteria. The modules of the other two networks were mainly dominated by Clostridia and Bacteroidia, with a loosened modular structure (Figure 6A).

In addition, 2 module hubs (nodes connected within a module) belonging to *Syntrophomonas* and *Sedimentibacter*, and 4 connectors (nodes linking different modules) belonging to *Anaerosalibacter*, *Tepidimicrobium*, *Caproiciproducens*, and *Sporanaerobacter*, were detected in the network of the acidic group (Figure 6C). For the network of the alkaline group, only 2 module hubs, *Tepidanaerobacter* and *DMER64*, were detected. Hence, it could be confirmed that networks grouped by pH exhibit distinct microbial network structures at the keystone node level.

### 3.6. The Assembly Process of Prokaryotic Communities in PMs

**Figure 7:**
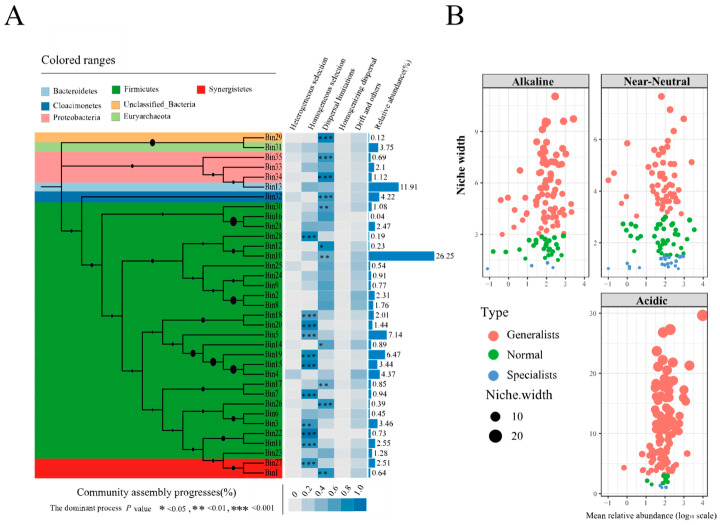
(**A**) The relative relevance and abundance of several ecological processes in each bin. (**B**) The niche width of prokaryotic communities in acidic, near-neutral, and alkaline PMs.

A total of 3911 OTUs were divided into 35 phylogenetic bins for revealing the assembly processes of prokaryotic communities based the iCAMP analysis, each of which was then analyzed separately as outlined [24] (Figure 7A). The results suggest that stochastic processes (62.07%, the average importance of 35 bins), including dispersal limitation (DL, 43.04%), homogenizing dispersal (HD, 1.45%), drift, and others (DR, 17.58%), contributed more than deterministic processes (37.93%), including homogeneous selection (HoS, 35.09%) and heterogeneous selection (HeS, 2.84%). HoS and DL dominated 14 and 20 bins, respectively, with 45.7% and 49.9% abundance in the total number of bins. Additionally, DR dominated 1 bin, with 4.4% abundance in the total number of bins. Significance was shown both for HoS and DL in 11 bins, respectively.

Firmicutes occupied 26 bins (72.94%, in the total abundance of bins), including 10 bins controlled by HoS (*p* < 0.05), 6 bins controlled by DL (*p* < 0.05), and 10 bins with no significant assembly processes. The other phyla (Synergistetes and Proteobacteria, etc.) occupied 9 bins (27.06%, in the total abundance of bins), including 5 bins controlled by DL (*p* < 0.05), 1 bin controlled by HoS (*p* < 0.01), and 3 bins with no significant assembly processes.

Niche width is the total number of resources utilized by a population in a community. In habitats where resources are shrinking, the niche width increases as populations expand their types of resources used to acquire sufficient resources. Conversely, the niche width decreases when resources become more available [32]. There were significant positive correlations between pH and niche width (Spearman’s = 0.583, *p* < 0.01). Generally, the microbial population of the PMs mainly consists of Generalists. Among the them, the near-neutral group has the highest number of Specialists (21) and the lowest number of Generalists (59). The acidic and alkaline groups have 107 and 85 Generalists and 4 and 5 Specialists, respectively (Figure 7B).

## 4. Discussion

As mentioned in the introduction, the quality (e.g., flavor and taste) of CSFB is mainly determined by the diversity and structure of prokaryotic communities in PMs, which are partly shaped by the physicochemical properties, especially the pH [33]. Therefore, this study systematically investigates the physicochemical properties, characteristics of prokaryotic community structures, diversities, and assembly processes across a pH gradient in PMs. This investigation is crucial for a deeper understanding of the regular patterns of assembly and variation in the prokaryotic community in PMs driven by pH, providing theoretical guidance for practical applications.

Previous studies have reported dozens of physicochemical properties, including pH, ${\mathrm{NH}}_{4}^{+},$ AP, TA, total nitrogen (TN), dissolved organic carbon (DOC), available K, humus/humic acid, ethanol, lactic acid, hexanoic acid, ${\mathrm{Ca}}^{2+},{\;\mathrm{Fe}}^{3+}$, etc., [4,15,34]. Among them, one or more parameters of the pH, AP, $\mathrm{TN}/{\mathrm{NH}}_{4}^{+},$ moisture, and TA/lactate/DOC were commonly identified as important indicators influencing microbial communities. This is why the above-mentioned physicochemical parameters (Figure 1) were selected for investigation in this work. To the best of our knowledge, this is the first specialized and systematic report elucidating the variations in physicochemical and prokaryotic information with changing pH in PMs. The pH scale of all tested PMs exhibited a gradient ranging from 3.46 to 8.85 (Figure 1). Significant correlations between other factors and pH were observed (Figure 1 and Table S1). Additionally, high contents of Mo and AN appeared in normal PMs with a pH ranging from 6 to 8. In addition, previous studies have reported pH as a strong abiotic factor influencing carbon availability [19], nutrient availability [35,36], and the solubility of metals [37]. All these indicated that the pH value could be used as an indicator to preliminarily assess the overall physicochemical properties of PMs.

### 4.1. pH as a Predictor of α- and β-Diversities of Prokaryotic Communities in PMs

To our knowledge, the pH exerts significant environmental pressure on microbial communities in fermentation processes (e.g., PMs, FGs, fermented vegetables) as well as in natural ecosystems such as soils and wetlands [15,27,38,39,40]. In this work, there were significant correlations between the pH and diversity indices that were observed (Figure 2). Among them, the ACE and Chao1 indices increased with pH rising, indicating that the species richness of the prokaryotic community increased appreciably from acidic to alkaline conditions. It can be confirmed by previous studies that the degraded and young PMs with lower pH have smaller species abundance (or Chao1 index) [12,15]. Furthermore, the theoretical maximum value of prokaryotic community diversity in PMs was observed at a pH value around 7, which was consistent with trends in soil [39]. Species diversity rapidly increased from pH 3 to 5 (Figure 2), indicating a drastic change in the prokaryotic community of PMs when pH ≤ 5. This could be confirmed by the result of a previous study, i.e., the prokaryotic community compositions changed drastically from 1-year PMs with pH average value of 3.57 to 10-year PMs with pH 5.00 [4]. Combining the results of the β-diversity analysis (Figure 3), we found that the change in prokaryotic communities in PMs, with a pH from 3.46 to 8.85, could be divided into three stages, including a stable period (pH ≤ 4 or pH ≥ 8), drastic change period (5 ≥ pH > 4), and relative stability period (8 ≥ pH > 5). Interestingly, these findings were aligned with results from studies on changes in bacterial and archaeal communities in various soils [39,41]. This significantly expanded our understanding of the rules governing α- and β-diversities of microbial communities across a pH gradient in natural fermentation systems. In summary, to some extent, the diversities of bacterial communities in different PMs across a wide pH range may be predictable. More importantly, these findings were conducive to accessing the PM quality and guiding actual CSFB production timely. For instance, the degraded PMs always have lower pHs (<5) [4,15,42], and the initial pH needs to be more than 5 when making the artificial PMs [13].

### 4.2. Variation Characteristics of Prokaryotic Community Structures and Biomarker Identification across a pH Gradient in PMs

Our findings also revealed a complex prokaryotic microbiota in PMs [12,15], with a total of 381 genera assigned into 27 phyla that were observed in this study. Many dominant prokaryotic taxa identified in our study were also detected and revealed in previous studies on PMs, including Firmicutes, Bacteroidetes, Proteobacteria, Euryarchaeota, Clostridia, Bacilli, Bacteroidia, *Lactobacillus*, *Caproiciproducens*, etc. [4,12,13,15,43]. This indicates that microbial taxa in PMs for CSFB production share a certain degree of similarity. This may due to the consistent CSFB production processes across China, which involve similar raw materials (e.g., sorghum, wheat, rice), long-term fermentation, anaerobic conditions, SSF fermentation with low pH values (about 3.6–4.4), and high ethanol content [11,27,44]. Recent studies have summarized the important functions of the complex prokaryotic microbiota in PMs into three aspects including: (i) forming flavor substances, (ii) adjusting the balance of microecology (i.e., microbial correlation network and physicochemical properties), and (iii) promoting the circulation of elements such as carbon, nitrogen, and sulfur in PMs [18,45]. Firstly, both the actual production experiences and theoretical research indicate that PM microorganisms contribute to the formation of flavor substances in CSFB. For instance, the PM microbiota significantly improves the contents of various flavor substances during a mimic experiment for CSFB production, especially volatile acids (13 kinds) and esters (37 kinds) [45]. Furthermore, various clostridial species (e.g., *Clostridium kluyveri*, *Clostridium tyrobutyricum*, *Caproicibacterium* sp., *Ruminococcaceae* bacterium CPB6, etc.) isolated or detected in PMs have a strong capacity to produce short-chain fatty acids (SCFAs) and medium-chain fatty acids (MCFAs), e.g., acetate, butyrate, caproate, etc. [9,10,46,47]. Caproate is considered a crucial fatty acid among them, primarily because it serves as the precursor to ethyl caproate, a key and abundant aroma compound in CSFB. Furthermore, the caproate is produced by PM clostridial species (e.g., *Clostridium* spp., *Caproicibacterium* spp.) using 2 biosynthetic pathways identified by using the substrates of lactate and ethanol, including reverse β-oxidation (RBO) and fatty acid biosynthesis (FAB) [48,49,50]. Moreover, PM microbiota can influence both bacterial and fungal communities in the CSFB fermentation system [45]. For example, a rapid increase in the population of lactic acid bacteria (LAB, such as *Lactobacillus*, *Lactococcus*, *Streptococcus*) may compromise PM microbiota stability [15]. *Caproiciproducens*, *Closiridium*_sensu_stricto_12, and other seven dominant genera with abundances in five or six groups of PMs were more than 1% (Table S2), indicating that these taxa may tolerate a wide pH range, which may play an important role in maintaining the stable pH of PM. For instance, *Petrimonas sulfuriphila* that interacted with *Caproicibacterium* sp., isolated from PMs, can adjust the pH of the fermentation system [51]. According to a phylogenetic analysis of the PM microbiota, various clostridial taxa could participate in the synthesis and degradation of carbon-, sulfur-, and nitrogen-containing compounds, e.g., some species of genera of *Clostridium*, *Caproicibacterium*, *Saccharofermentans*, *Gracilibacter*, *Syntrophomonas*, *Anaerovorax*, *Alkalibaculum*, *Sedimentibacter*, *Dethiobacter*, *Desulfosporosinus*, etc., having an exceptionally broad ability to ferment various substrates (e.g., carbohydrates, proteins, polypeptide, thiosulfate, etc.) [18,49]. In addition, the following microorganisms were also detected in the tested PMs, including methanogens including hydrogenotrophs (*Methanobacterium*, *Methanoculleus*, and *Methanobrevibacter*), acetotrophs (*Methanosaeta*), and the genus Methanosarcina, which could utilize H_2_, acetate, methanol, and methylamine, respectively [15,52,53]. Thus, the ability to predict and monitor the composition of PM microbial communities promptly and easily is crucial for revealing the functions of complex microbiota and assessing PM qualities.

The variation characteristics of structures and diversities of the prokaryotic community in PMs were consistent (Figure 4). Three distinct periods as mentioned earlier were more clearly observed in the changing process of prokaryotic community structure with increasing pH (Figure 3). During the stable period (pH ≤ 4), the community structures in PMs were more similar and only four dominant prokaryotes, including *Acinetobacter*, *Caproiciproducens*, *Clostridium_*sensu_stricto_12, and *Lactobacillus* were detected (Figure 4 and Figures S3–S5). *Lactobacillus* predominated (76.1%), resembling the composition found in young and degenerated PMs [4,14]. This dominance is attributed to the enrichment of acid-resistant microorganisms (i.e., *Lactobacillus*) that could be easily enriched, in contrast to those with optimal growth at a pH near neutral (e.g., Clostridia, Methanobacteria, Methanomicrobia, etc.) [15]. Simultaneously, the accumulations of lactate and bacteriocins (e.g., nisin and lactacin) can directly or indirectly inhibit the growth and reproduction of various microorganisms, contributing to the dominance of *Lactobacillus* [3,54]. During the drastic change period (4 < pH ≤ 5), the number of dominant prokaryotes increased to 9, with rapid increases in the abundances of Methanobacteria, Methanomicrobia, Synergistia, Bacteroidia, and Clostridia. Additionally, the total abundance of other genera, each with relative abundances less than 1%, rose from 12.36% to 43.61%. In contrast, the abundance of Bacilli, primarily constituted by *Lactobacillus*, sharply declined from 77.23% to 26.38% compared to PMs with pH ≤ 4 (Figure S5). Interestingly, the prokaryotic community structures in PMs showed significant differences resulting in a dispersed distribution of PMs (4 < pH ≤ 5) across groups A to F (Figure 3 and Figure S5). This pattern resembled the β-diversity results observed in various soils, as documented in previous studies [39,41], suggesting similarities in community structure dynamics [39,41]. The complex prokaryotic microbial community with higher species diversity is constantly formed mainly during this period. For the relative stability period, the dominant phyla (i.e., Firmieutes, Baeteroidetes, Euryarchaeota, Synergistetes) and genera (e.g., *Caproieiproducens*, *Methanoculleus*, *Syntrophomonas*, *Aminobacterium*, *Sedimentibacter*, *Clostridium*_sensu_strieto 12, *Petrimonas*, *Proteiniphilum*, etc.) in PMs were also detected in old and high-quality PMs, and some dominant genera play important roles in producing volatiles and balancing the microecology, e.g., *Caproieiproducens*, *Methanoculleus*, *Syntrophomonas*, *Petrimonas*, *Sedimentibacter*, etc. [4,7,12,18]. All these indicated that the prokaryotic communities in PMs (5 < pH ≤ 8) could be used for producing high-quality CSFB, which can be confirmed by CSFB production experience [15,55]. For another stable period (pH > 8), the structures of the prokaryotic community were similar to those in PMs with a pH from 5 to 8, but the relative abundances of *Hydrogenispora* and *Syntrophomonas* increased, while those of *Caproiciproducens*, *Lactobacillus*, *Closiridium*_sensu_stricto_12, and other related taxa decreased. In addition, the distribution of PMs in this stage becomes more concentrated (Figure 3), which was similar to that of PMs with a lower pH (pH ≤ 4), indicating that extreme pH conditions could form the more similar structures of prokaryotic communities in PMs.

The changes in biomarkers (e.g., *Lactobacillus*, *Acinetobacter*, *Methanoculleus*, *Syntrophaceticus*, *Sporanaerobacter*, etc.) in different groups of PMs were also observed (Figure 5), indicating that the prokaryotic preferences for specific habitats and potential functions varied with pH. For instance, *Lactobacillus* serves as a biomarker for group A of PMs and could be enriched in fermentation systems with a lower pH (3.54–3.92), primarily due to its acid resistance [56].

### 4.3. Effect of pH on the Network Characteristics of Prokaryotic Communities in PMs

In the natural environment, microbial community evolution is influenced by both environmental factors and inter-microbial interactions [24]. By mapping the co-occurrence networks of samples from different pH conditions, we investigated the co-occurrence patterns of microorganisms to explore succession patterns of pit mud prokaryotic communities. In co-occurrence networks, positive correlations indicate either a niche overlap or positive interactions, whereas negative correlations denote dissimilar niches or negative interactions [3]. The results indicated that the topological structure of the prokaryotic community network became simpler as the pH value approached neutrality. The near-neutral network had fewer nodes and edges, the modules were looser from each other, and no network node was recognized as a keystone. A potential symbiotic relationship between Clostridia and Bacteroidia, Methanomicrobia, and Methanobacteria was found in the near-neutral network. Additionally, there was a lack of clear boundaries between acidic network modules. Recent reports indicate that module boundaries are unclear in PM networks aged 10–50 years, whereas clear module boundaries are observed in PM networks exceeding 100 years. Module boundaries were once again blurred in alkaline networks, and more pairwise relationships emerged. As batch-to-batch fermentation cycles increased, microorganisms in pit mud (PM) were influenced by various environmental pressures and complex microbial interactions. Those not adapted to the environment would be filtered out, leading to the formation of a simplified and modular co-occurrence network. Our results suggest that pH may accelerate this process as an environmental factor. This had important implications for the aging of PM.

### 4.4. The Assembly Mechanisms of Prokaryotic Communities in PMs Based on iCAMP

However, characterizing such complex communities, quantifying the accompanying ecological processes, and dissecting the mechanisms controlling biodiversity and community composition are extremely challenging [24]. Various ecological processes, like natural selection, occur at more specific biological levels, such as genotype and population, rather than the community as a whole. Consequently, it could be more practical to conduct research at the finer taxonomic level, which was the reason for selecting iCAMP as the preferred framework (Figure 6A).

The results were similar to those of related studies, emphasizing the dominance of stochastic processes in the community assembly processes, while also confirming the important role of deterministic processes [30]. Overall, the microbial community assembly process of PMs was affected by the combined effects of stochastic and deterministic processes across the pH range of 3 to 9. Among them, the influence of stochastic processes was slightly larger, accounting for 57.2%, and the effect of deterministic accounted for 42.8%. To explain further, the assembly process was influenced mainly by DL (35.93%) and HoS (41.35%). The environmental characteristics exert selective pressure on the assembly of the microbial community in PM. Intuitively, under the extreme acidic or alkaline pH conditions, this would lead to the assembly of phylogenetically more clustered communities through deterministic processes. In contrast, pH conditions close to neutral lead to phylogenetically less clustered bacterial communities with more stochasticity. Interestingly, some bins produced similar results to our predictions (such as bin4), while others deviated from our expectations. This also illustrates that a finer classification of phylogenetic signals can help us understand the assembly process of microbial flora.

The decreasing robustness of the PM microbial community was mainly due to the disproportionate abundance between *Caproiciproducens* and *Lactobacillus*; *Lactobacillus* has greater adaptive capacity; *Caproiciproducens* primarily concentrated in bin4, bin15, and bin19 (Figure S7). Bin4 is mainly influenced by HoS when pH < 5, and by DL and DR when pH > 5. In recent research, the relative influence of HoS was stronger in highly acidic soils, whereas the stochastic process was stronger in soils closer to a neutral pH. Bin15 and 19 were primarily influenced by HoS across all pH levels. These bins belong to the same genus, *Caproiciproducens*, but exhibit a different assembly model under the impact of pH. Choosing a culture with a broader ecological niche could streamline the design process of synthetic microbial communities by offering more versatility and resilience. In the future, it may be feasible to isolate strains with high caproic acid production and robust pH tolerance, potentially enabling the engineering of caproic acid bacteria with enhanced acid-resistant properties.

## 5. Conclusions

This study focused on the relationship between pH and the prokaryotic community in PMs. It was found that a lower-pH habitat resulted in a significant reduction in microbial diversity. Of the eleven dominant genera showing significant correlations with pH, eight exhibited a decrease in abundance in response to pH changes, indicating their sensitivity to pH. The network analysis results suggested that pH as an environmental factor may expedite microbial ecological cluster aggregation in PM. Using iCAMP, a method for assessing prokaryotic community assembly processes, a combined influence of stochastic and deterministic processes was revealed. Stochastic processes emerged as the primary driver in shaping the community structure.

## Data Availability

The sequencing data were submitted to the Sequence Read Archive (SRA) of the NCBI database under BioProject: PRJNA1025237.

## Supplementary Materials

The following supporting information can be downloaded at: https://www.mdpi.com/article/10.3390/foods13081196/s1. Figure S1: (A) Pit mud samples were collected from Henan province, China. (B) Sampling locations of pit muds in the fermentation pit; Figure S2: Rarefaction curve was shown all pit mud samples; Figure S3: Relative abundance for all pit mud samples at phylum level; Figure S4: Relative abundance for all pit mud samples at class level; Figure S5: Relative abundance for all pit mud samples at genus level; Figure S6: The results of Linear Discriminant Analysis (LDA) effect size (LEfSe) algorithm. The cutoff LDA score was 4.0; Figure S7: Relative importance of various assembly processes in the compartment of bins under equal pH gradient grouping; Table S1: Spearman’s correlation was calculated among physicochemical properties; Table S2: Relative abundance of the prokaryotes in PMs at the genus level; Table S3: Samples grouping and size information; Table S4; The topology of networks.
