# Supplementary material for: Illuminating the Characteristics and Assembly of Prokaryotic Communities across a pH Gradient in Pit Muds for the Production of Chinese Strong-Flavor Baijiu
Source: Foods. 2024 Apr 15;13(8):1196. doi: 10.3390/foods13081196 (PMC11048939; doi:10.3390/foods13081196)
Supplement: Supplementary file 1 [file foods-13-01196-s001.zip › Supplemental Figures.pdf]

## Supplemental figure captions

**Figure S1** （A）Pit mud samples were collected from Henan province, China. (B) Sampling locations of pit muds in the fermentation pit.

**Figure S2** Rarefaction curve was shown all pit mud samples.

**Figure S3** Relative abundance for all pit mud samples at phylum level.

**Figure S4** Relative abundance for all pit mud samples at class level.

**Figure S5** Relative abundance for all pit mud samples at genus level.

**Figure S6** The results of linear discriminant analysis (LDA) effect size (LEfSe) algorithm. The cutoff LDA score was 4.0.

**Figure S7** Relative importance of different assembly processes within bins grouped by equal pH gradient.

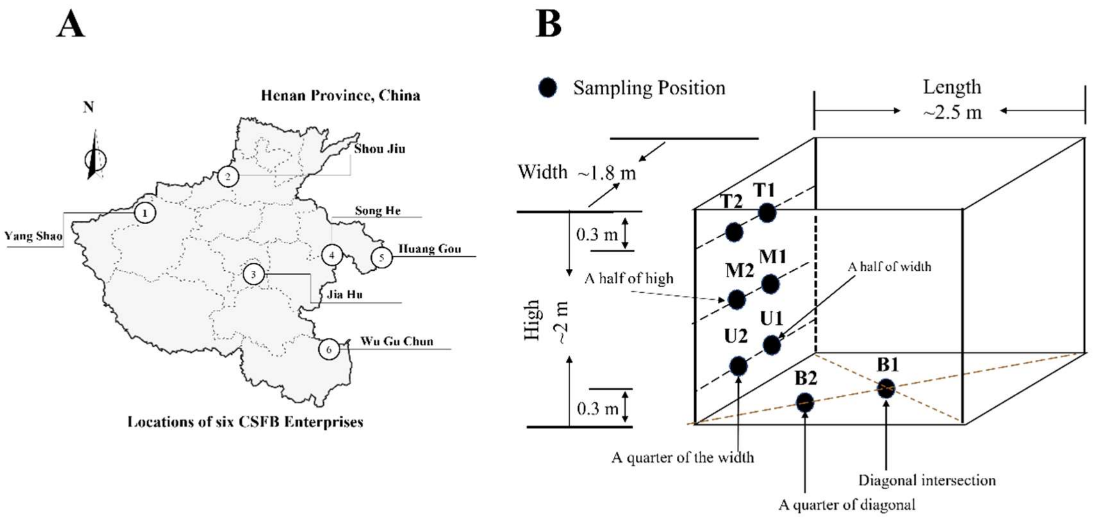


**Figure S1**

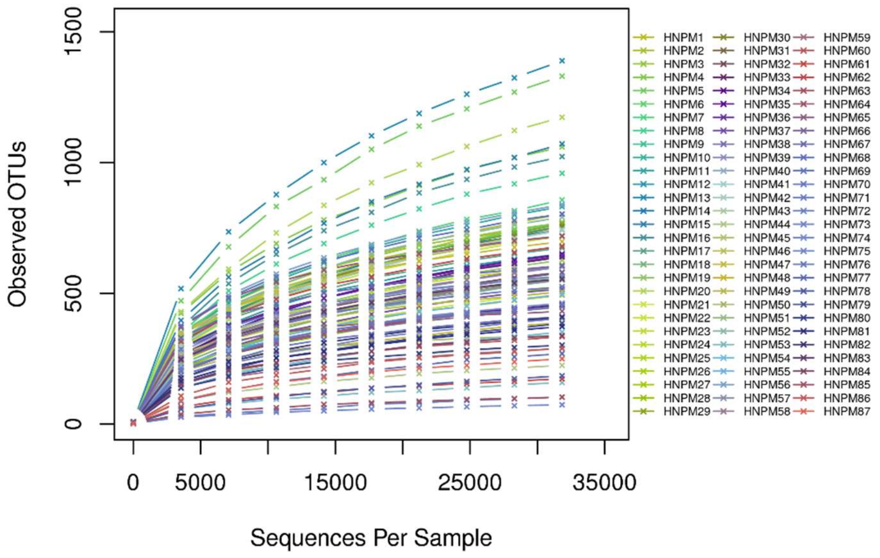

Observed OTUs
Sequences Per Sample
0
500
1000
1500
0
5000
15000
25000
35000


**Figure S2**

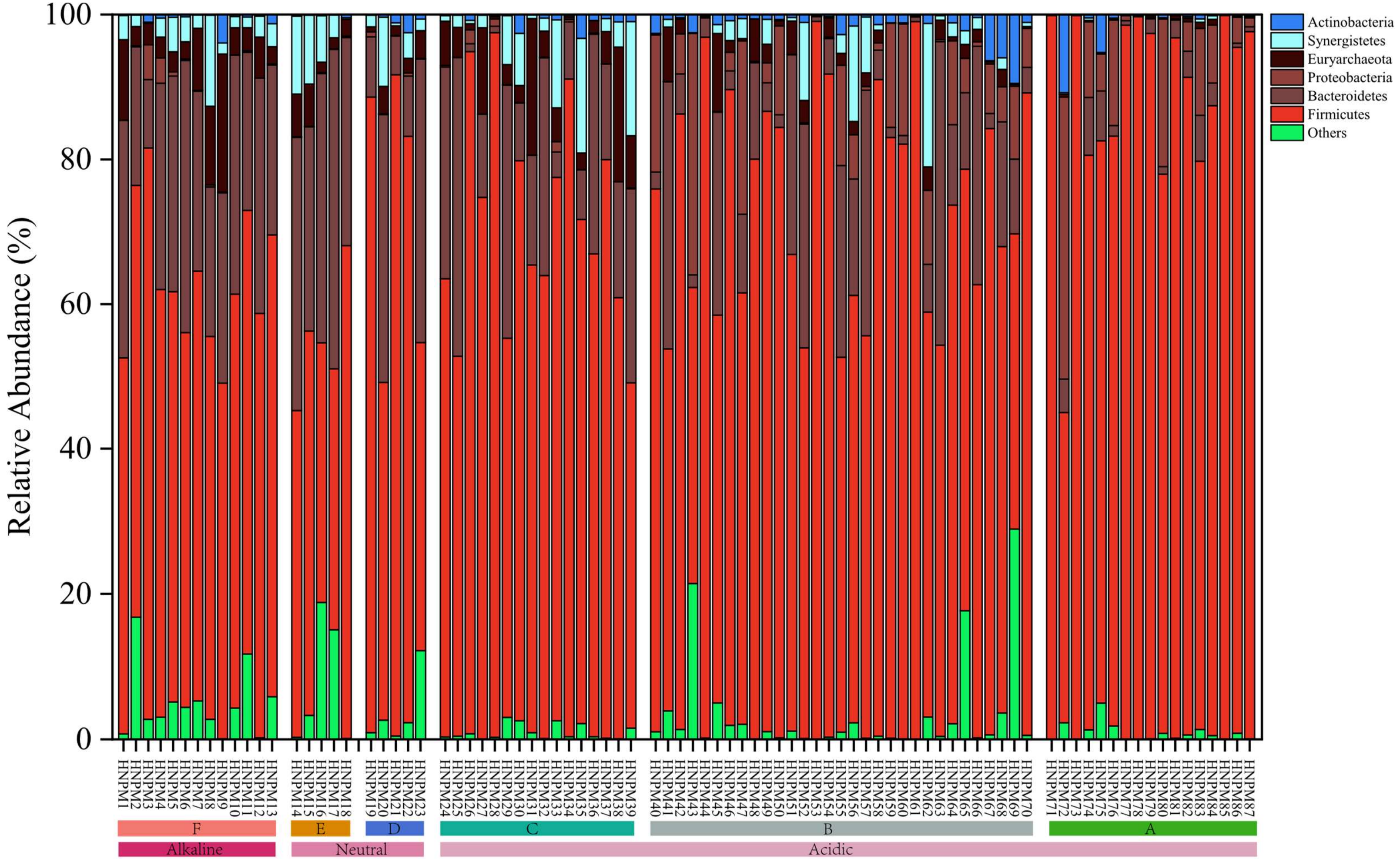

Relative Abundance (%)
100
80
60
40
20
0
Actinobacteria
Synergistetes
Euryarchaeota
Proteobacteria
Bacteroidetes
Firmicutes
Others
F
E
D
C
B
A
Alkaline
Neutral
Acidic


**Figure S3**

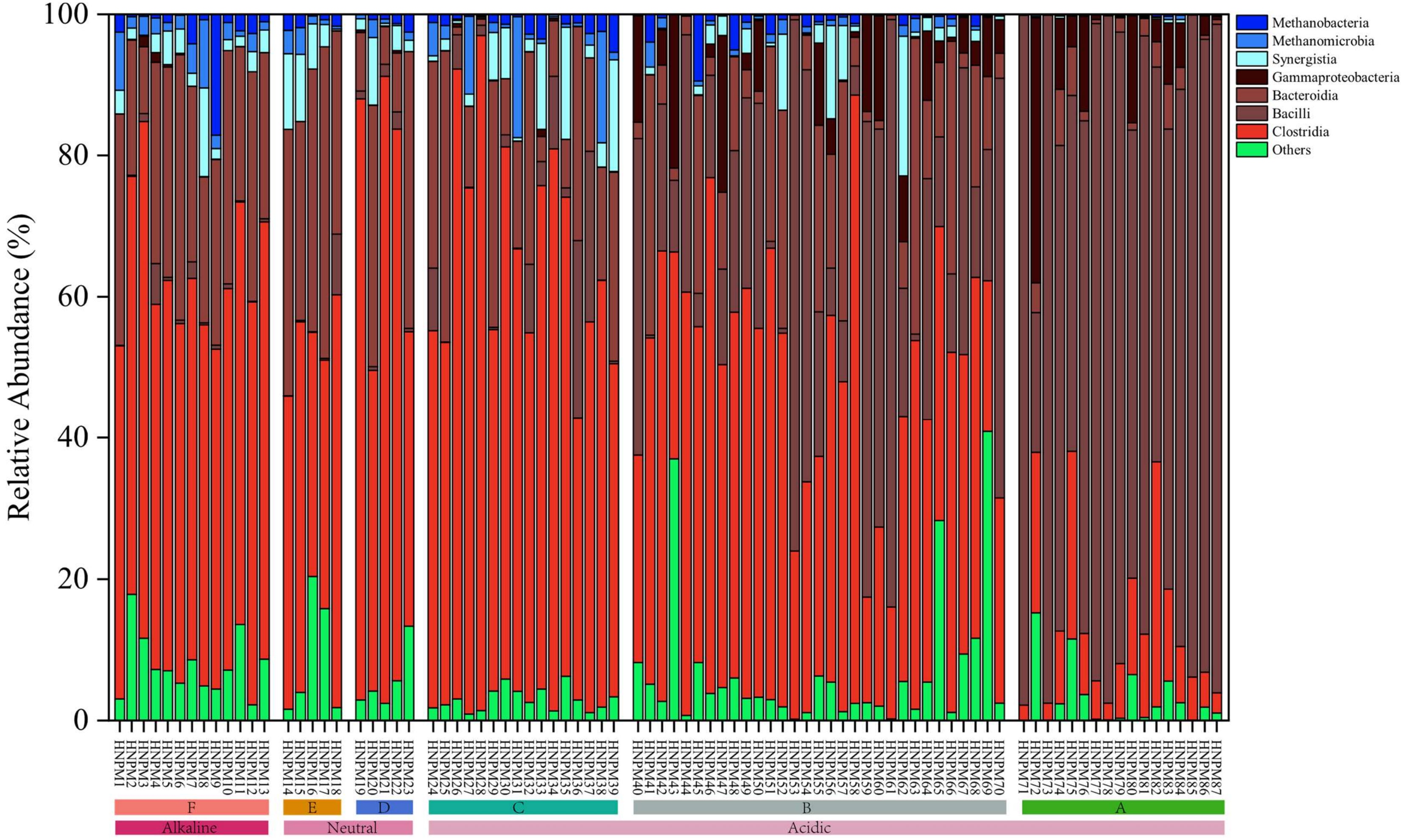

Relative Abundance (%)
100
80
60
40
20
0
Methanobacteria
Methanomicrobia
Synergistia
Gammaproteobacteria
Bacteroidia
Bacilli
Clostridia
Others
F
E
D
C
B
A
Alkaline
Neutral
Acidic


**Figure S4**

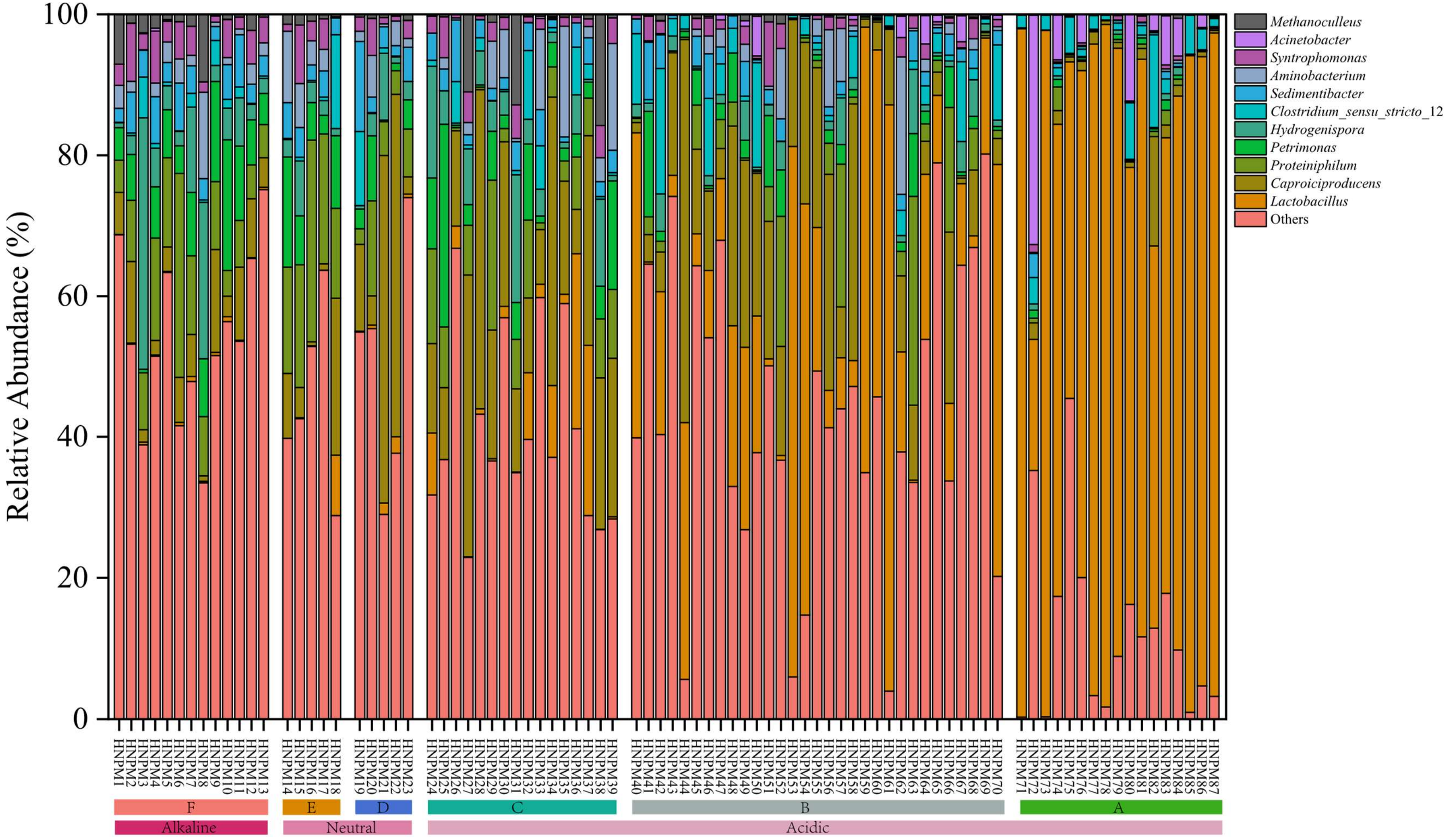
Relative Abundance (%)
0
20
40
60
80
100
Methanoculleus
Acinetobacter
Syntrophomonas
Aminobacterium
Sedimentibacter
Clostridium_sensu_stricto_12
Hydrogenispora
Petrimonas
Proteiniphilum
Caproiciproducens
Lactobacillus
Others
F
E
D
C
B
A
Alkaline
Neutral
Acidic

**Figure S5**

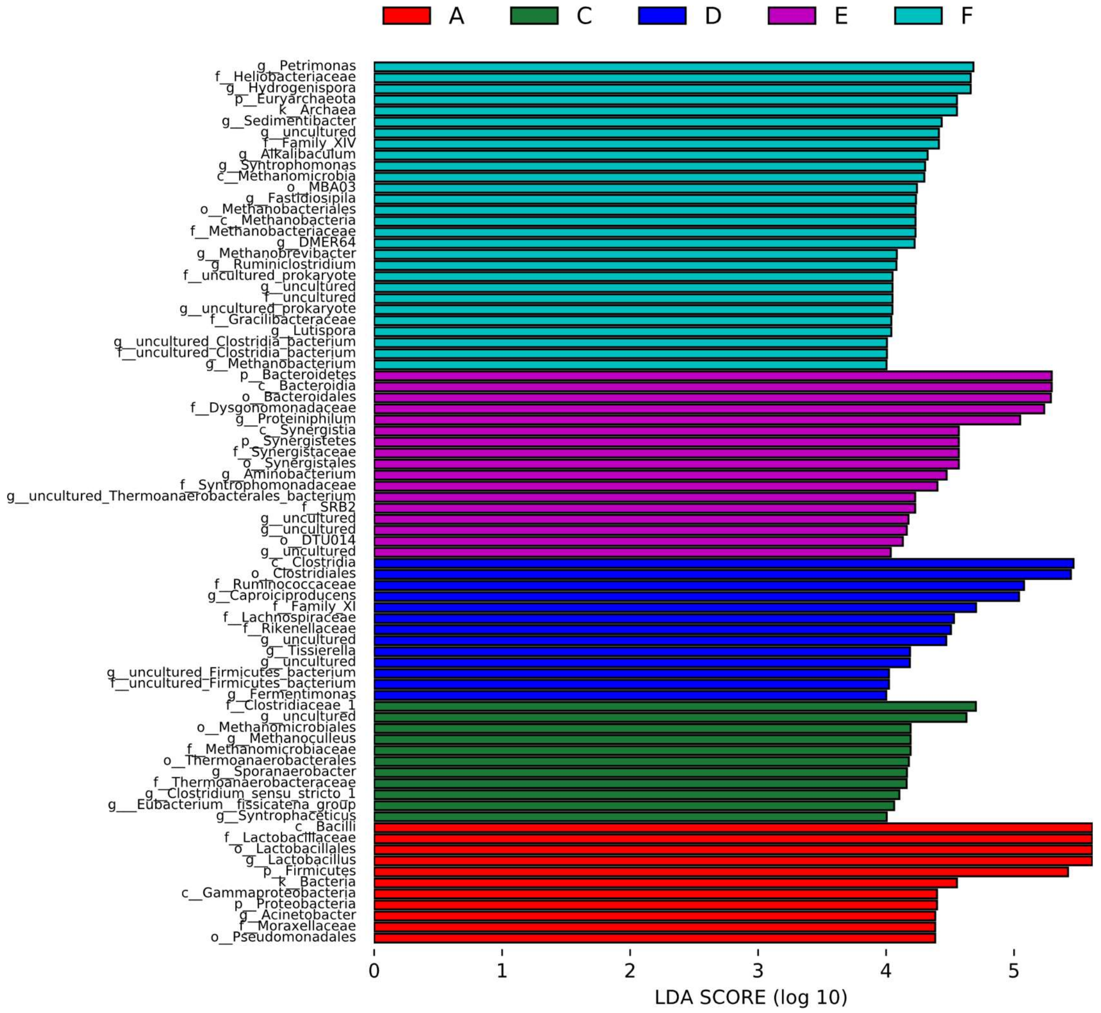

A
C
D
E
F
g__Petrimonas
f__Heliobacteriaceae
g__Hydrogenispora
p__Euryarchaeota
k__Archaea
g__Sedimentibacter
g__uncultured
f__Family_XIV
g__Alkalibaculum
g__Syntrophomonas
c__Methanomicrobia
o__MBA03
g__Fastidiosipila
o__Methanobacteriales
c__Methanobacteria
f__Methanobacteriaceae
g__DMER64
g__Methanobrevibacter
g__Ruminiclostridium
f__uncultured_prokaryote
g__uncultured
f__uncultured
g__uncultured_prokaryote
f__Gracilibacteraceae
g__Lutispora
g__uncultured_Clostridia_bacterium
f__uncultured_Clostridia_bacterium
g__Methanobacterium
p__Bacteroidetes
c__Bacteroidia
o__Bacteroidales
f__Dysgonomonadaceae
g__Proteiniphilum
c__Synergistia
p__Synergistetes
f__Synergistaceae
o__Synergistales
g__Aminobacterium
f__Syntrophomonadaceae
g__uncultured_Thermoanaerobacterales_bacterium
f__SRB2
g__uncultured
g__uncultured
o__DTU014
g__uncultured
c__Clostridia
o__Clostridiales
f__Ruminococcaceae
g__Caproiciproducens
f__Family_XI
f__Lachnospiraceae
f__Rikenellaceae
g__uncultured
g__Tissierella
g__uncultured
g__uncultured_Firmicutes_bacterium
f__uncultured_Firmicutes_bacterium
g__Fermentimonas
f__Clostridiaceae_1
g__uncultured
o__Methanomicrobiales
g__Methanoculleus
f__Methanomicrobiaceae
o__Thermoanaerobacterales
g__Sporanaerobacter
f__Thermoanaerobacteraceae
g__Clostridium_sensu_stricto_1
g___Eubacterium__fissicatena_group
g__Syntrophaceticus
c__Bacilli
f__Lactobacillaceae
o__Lactobacillales
g__Lactobacillus
p__Firmicutes
k__Bacteria
c__Gammaproteobacteria
p__Proteobacteria
g__Acinetobacter
f__Moraxellaceae
o__Pseudomonadales
0
1
2
3
4
5
LDA SCORE (log 10)


**Figure S6**

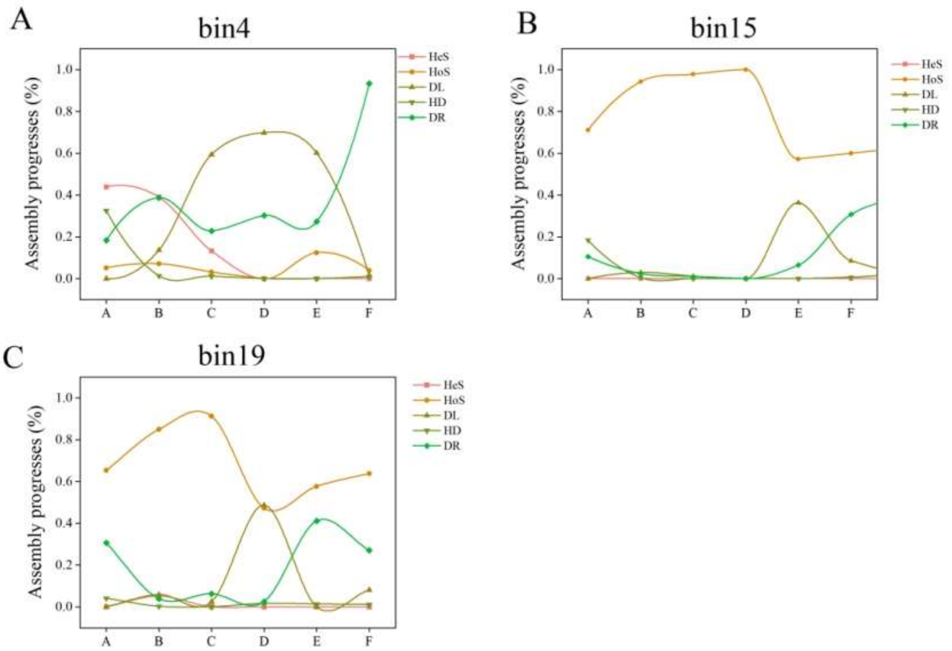
A
bin4
B
bin15
C
bin19
Assembly progresses (%)
HeS
HoS
DL
HD
DR
A
B
C
D
E
F

**Figure S7**
