# Supplementary material for: Illuminating the Characteristics and Assembly of Prokaryotic Communities across a pH Gradient in Pit Muds for the Production of Chinese Strong-Flavor Baijiu
Source: Foods. 2024 Apr 15;13(8):1196. doi: 10.3390/foods13081196 (PMC11048939; doi:10.3390/foods13081196)
Supplement: Supplementary file 1 [file foods-13-01196-s001.zip › Supplementary Tables.pdf]

Table S1. Spearman's correlation was calculated among physicochemical properties.

	pH	TA	AN	AP	Mo
pH	1				
TA	-0.912**	1			
AN	0.690**	-0.717**	1		
AP	0.254*	-0.176	0	1	
Mo	0.213*	-0.099	0.502**	0.056	1
sample size	87	87	87	87	87

* Spearman's $P < 0.05$

** Spearman's $P < 0.01$

Table S2. Relative abundance of the prokaryotes in PMs at the genus level

**group**	**A**	**B**	**C**	**D**	**E**	**F**
**subgroup**	**acidic group**	**acidic group**	**acidic group**	**Near-neutral group**	**Near-neutral group**	**alkaline group**
Lactobacillus	76.12	22.86	5.45	1.02	1.76	0.31
Caproiciproducens	1.88	14.26	20.68	23.39	7.46	6.04
Proteiniphilum	0.59	4.33	6.88	6.13	18.47	9.73
Petrimonas	0.42	2.59	6.19	3.49	8.17	7.77
Hydrogenispora	0.32	1.92	5.51	3.32	3.25	7.86
*Clostridium*_sensu_stricto_12	3.11	3.72	2.28	2.31	2.96	1.27
Sedimentibacter	0.44	2.12	3.17	4.86	3.51	4.21
Aminobacterium	0.11	2.17	4.07	2.50	4.58	2.61
Syntrophomonas	0.29	1.44	2.29	2.28	3.31	3.93
Acinetobacter	4.26	0.70	0.03	0.02	0.01	0.07
Methanoculleus	0.09	0.27	2.78	0.50	0.96	2.27
Others	9.37	27.53	23.67	26.72	19.86	28.83
Unclassified	2.98	16.08	17.00	23.47	25.69	25.06

Table S3. Samples grouping and size information.

**group**	**A**	**B**	**C**	**D**	**E**	**F**
subgroup	acidic group	acidic group	acidic group	near-neutral group	near-neutral group	alkaline group
pH range	4>pH≥3	5>pH≥4	6>pH≥5	7>pH≥6	8>pH≥7	9>pH≥8
sample size	16	31	16	5	4	14

Table S4. The topology of co-occurrence networks

	**Acidic group**	**Near-neutral group**	**Alkaline group**
Node	434	113	210
Edge	4727	118	288
Average Weight Distance	15.04	2.03	2.54
Diameter	9	7	10
Average Path Length	3.458	1.722	3.677
Density	0.05	0.02	0.01
Modularity index	0.64	0.94	0.87
